# Gender differences in the long-term effects of a nutritional intervention program promoting the Mediterranean diet: changes in dietary intakes, eating behaviors, anthropometric and metabolic variables

**DOI:** 10.1186/1475-2891-13-107

**Published:** 2014-11-22

**Authors:** Vicky Leblanc, Catherine Bégin, Anne-Marie Hudon, Marie-Michelle Royer, Louise Corneau, Sylvie Dodin, Simone Lemieux

**Affiliations:** Institute of Nutrition and Functional Foods, Laval University, 2440 Hochelaga Boulevard, Québec, G1V 0A6 Canada; School of Psychology, Laval University, Pavillon Félix-Antoine Savard, 2325 rue des Bibliothèques, Québec, G1V 0A6 Canada; Department of Obstetrics and Gynaecology, Laval University, Pavillon Ferdinand-Vandry, 1050 Medicine Avenue, Québec, G1V 0A6 Canada

**Keywords:** Gender differences, Mediterranean diet, Cardiovascular risk, long-term dietary changes

## Abstract

**Background:**

Long-term adherence to principles of the Mediterranean diet (MedDiet) following a nutritional intervention promoting the Mediterranean food pattern in Canadian men and women is not known. Moreover, gender differences in dietary and metabolic profile in such an intervention context has never been addressed. Objective was to determine gender differences in long-term effects of a 12-week nutritional intervention program promoting the adoption of the MedDiet and based on the Self-Determination Theory (SDT) on dietary intakes, eating behaviors, anthropometric and metabolic variables, in men and women presenting cardiovascular risk factors.

**Methods:**

Sixty-four men and 59 premenopausal women were recruited. The 12-week nutritional program used a motivational interviewing approach and included individual and group sessions. A food frequency questionnaire was administered to evaluate dietary intakes from which a Mediterranean score (Medscore) was derived and the Three-Factor Eating Questionnaire allowed assessment of eating behaviors. Measurements were performed at baseline and after the 12-week nutritional intervention, and then at 3 and 6-month post intervention.

**Results:**

No gender difference was observed in changes in the Medscore during the nutritional intervention and follow-up. However, the Medscore returned towards baseline values during follow-up in men and women (*P* <0.0001). Men reported larger decreases in red and processed meat and larger increases in whole fruit intakes than women (*P* = 0.03 and *P* = 0.04, respectively). Men showed a greater decrease in habitual susceptibility to disinhibition than women (*P* = 0.03). A gender by time interaction was found for waist circumference, i.e. men had lower waist circumference at the end of the intervention as well as at follow-up than at baseline while women’s waist circumference decreased in response to the intervention only (*P* = 0.05). As for metabolic variables, changes observed in total-cholesterol (C) to HDL-C ratio, triglyceride levels and triglycerides to HDL-C ratio were more pronounced in men than in women after the intervention as well as at follow-up (*P* ≤0.03).

**Conclusions:**

Our results indicate that the 12-week nutritional intervention based on the SDT leads to more pronounced beneficial changes in long-term dietary intakes in men than in women and to greater improvements in metabolic profile in men.

**Trial registration:**

Current Controlled Trials NCT01852721.

## Introduction

Evidence of the benefits of the Mediterranean diet (MedDiet) on health is now well established in the literature. Indeed, the MedDiet is recognized as one of the best models of health food patterns providing protection against chronic diseases, such as cardiovascular diseases (CVD) and cancer [1, 2]. Accordingly, high level of adherence to the MedDiet has been reported to be negatively associated with several cardiovascular risk factors although heterogeneity related to demographic characteristics (i.e., country, gender, socio-cultural status) has been observed [3].

Changing dietary habits represents a major challenge for many people [4, 5]. In this regard, although adherence to the MedDiet is beneficial for CVD prevention [5, 6], previous studies reported difficulties regarding maintenance of the MedDiet principles [5, 7]. Evidence suggests favourable diet adherence outcomes in the context of different intervention settings lasting over a 12-month period [8] and including sustained support to individuals in the long term (e.g., information, individual or group sessions) [9, 10]. These results therefore indicate the relevance of providing long-term support to individuals. However, according to actual public health priorities in Canada [11], long-term nutritional support, which requires several professional and financial resources, remains unrealistic to address CVD prevention at a population level. In this context, nutritional interventions supporting autonomy and competency of individuals, in agreement with the Self-Determination Theory (SDT) which emphasizes the importance of motivation quality and self-determined forms of motivation towards behavior change [12], are relevant.

Beyond intervention modalities, a variety of individual factors have been proposed to influence the ability to maintain dietary changes. In fact, individual preferences in food selection, preparation and consumption, variables related to socioeconomic and education levels and lifestyle factors such as the degree of engagement in physical activity have been suggested to play a role in the ability to maintain dietary changes in the longer term [13, 14]. Moreover, previous studies have identified gender as a key determinant of food choices. This can be explained by differences between men and women in attitudes, beliefs and motivation towards healthy eating and also in their awareness of diet and health issues [15–17], which can possibly influence level of adherence to dietary recommendations. In the context of the MedDiet, a higher success in improving adherence to the MedDiet was observed among men than women after one year of follow-up in the PREDIMED trial, which includes Spanish men and women presenting high risk for CVD [9]. On the other hand, a study measuring the impact of a MedDiet education program in hypercholesterolemic men and women from the Netherlands showed that whereas women improved their dietary intakes in accordance with the education program and significantly decreased their total-cholesterol (C) levels, no such change was observed in men [5]. These differences between men and women suggest that the process of sustaining dietary changes following a nutritional intervention promoting the adoption of the MedDiet could be influenced by gender, whether or not individuals come from Mediterranean regions. The feasibility of adopting and maintaining the MedDiet has been reported in Canadian women [18], but no data are available for Canadian men.

Evidence also indicates that the impact of adhering to dietary recommendations on anthropometric and metabolic profiles can be modulated by sex and gender differences. Indeed, metabolic changes in response to modification in dietary intakes can be explained in part by sex differences, which essentially refer to biological and physiological characteristics that distinguish males from females [19], such as sex hormones. Accordingly, a study published by our team showed improvement in insulin homeostasis (i.e. insulin concentrations 2 hours after an oral administration of 75 g of glucose) in men but not in women, in response to a 4-week MedDiet provided in the context of a controlled study where all food and drinks were provided to participants in isoenergetic conditions [20]. On the other hand, gender differences previously reported in eating behaviors (e.g., in dietary restraint and disinhibition levels) [21] could influence, through its association with dietary intakes [22], the level of adherence to dietary recommendations in men and women and therefore could also influence long-term changes in anthropometric and metabolic variables.

Overall, although the adoption of the MedDiet is recognized for its benefits on metabolic profile, the long-term adherence to principles of the MedDiet following a nutritional intervention program promoting the Mediterranean food pattern in Canadian men and women is not known. Moreover, gender differences in dietary intakes, eating behaviors, anthropometric variables and metabolic profile in such an intervention context have never been addressed. The objective of this study was therefore to determine gender differences in long-term effects of a 12-week nutritional intervention program promoting the adoption of the MedDiet and based on the SDT on dietary intakes, eating behaviors, anthropometric and metabolic variables, in Canadian men and women presenting with risk factors for CVD.

## Methodology

### Participants

This study was conducted among a sample of 64 men and 59 premenopausal women aged between 25 and 50 years old, and recruited through different media advertisements in the Québec City Metropolitan area, Canada. In women, a follicle-stimulating hormone (FSH) measurement was performed if needed (e.g., when women presented periods irregularities) to confirm the premenopausal status (FSH <20 IU/l) [23]. Men and women had to present slightly elevated LDL-C concentrations (between 3.0 and 4.9 mmol/l) [24] or a total-C to HDL-C ratio ≥5.0, and at least one of the four following criteria of the metabolic syndrome [25] : 1) triglyceride concentrations ≥1.7 mmol/l; 2) fasting glycaemia between 6.1 et 6.9 mmol/l; 3) blood pressure concentrations ≥130/85 mm Hg; 4) waist circumference ≥80 cm in women and ≥94 cm in men [26]. Participants also had to have a stable body weight (±2.5 kg) for a minimum of three months prior to the beginning of the study and to be implicated in food purchases and/or preparation at home. We excluded men and women who had cardiovascular events and who used medication that could affect dependent variables under study. Pregnant women, smokers, participants with an alcoholism history or with a high Mediterranean score (Medscore >29, i.e. food pattern already highly concordant with the MedDiet) [6] were also excluded. All subjects voluntarily agreed to participate in the research project and written informed consent was obtained from all men and women prior to their participation in the study. This study was approved by the Laval University Research Ethics Committee.

### Study design

**Figure 1:**
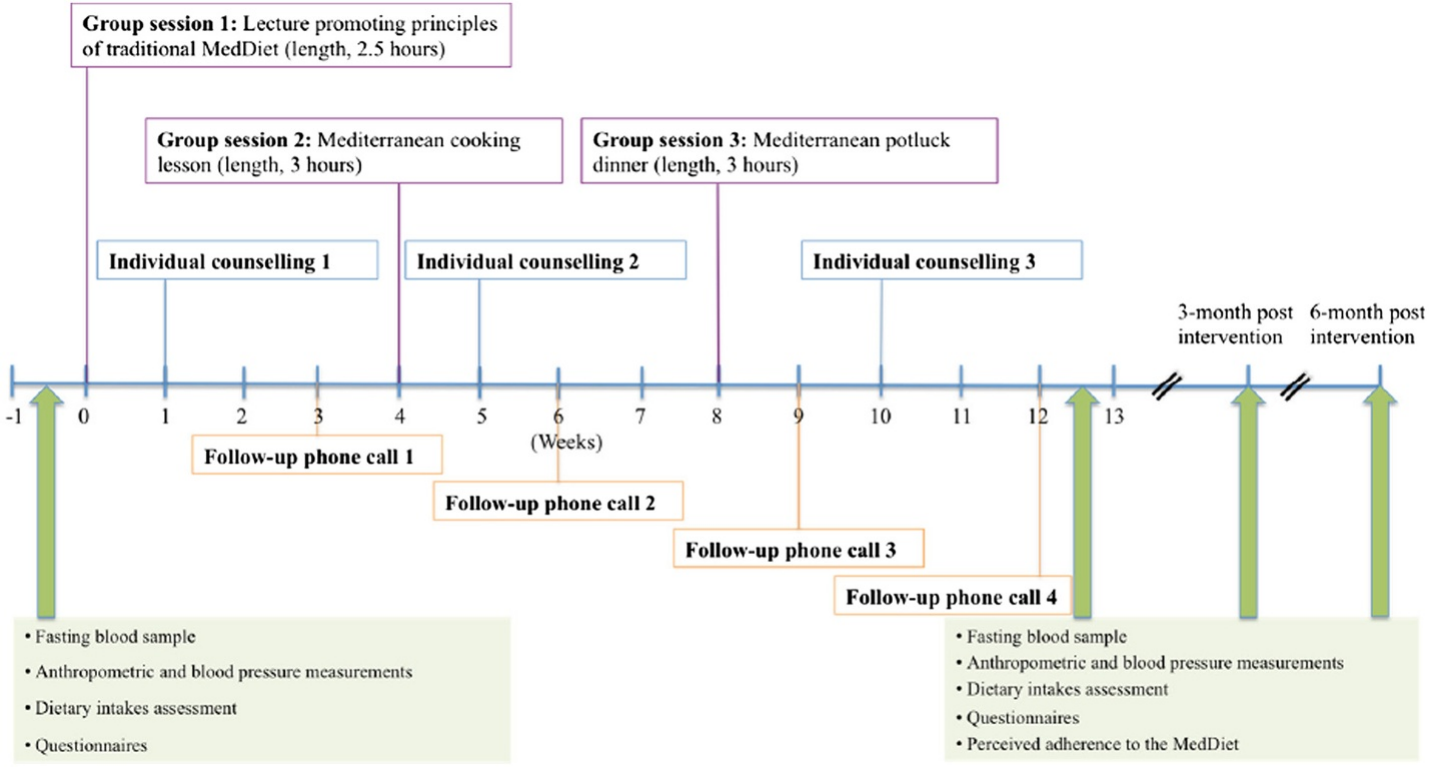
**Description of the 12-week nutritional intervention program and measurements performed at baseline (T = 0), after the end of the intervention (T = 3 months) and then at 3-month and 6-month post intervention (T = 6 months and T = 9 months respectively).**

The 12-week nutritional program was based on the Self-Determination Theory (SDT) and used a motivational interviewing (MI) approach. Briefly, the study was conducted in five phases (from January 2010 to November 2012) and the nutritional intervention included three group sessions (10–15 individuals/session), three individual sessions and four follow-up phone calls with a registered dietitian (Figure 1). Three registered dietitians were trained to provide a standardized intervention and participants always met with the same dietitian during individual sessions. The first group session was a lecture, provided by the same dietitian in all groups, and aimed at explaining principles of the traditional MedDiet. The second group session was a Mediterranean cooking lesson during which men and women had to cook a Mediterranean meal. Then, during the third group session participants had to share a Mediterranean potluck dinner aimed at discussing barriers in adopting dietary recommendations which occurred during the intervention. Individual counselling took place at weeks one, five and 10 and lasted between 45 minutes and one hour for each appointment. Individual follow-up phone calls took place at weeks three, six, nine and 12, and lasted for about 20–30 minutes for each phone call. The main objective of individual counselling and follow-up phone calls was to assess dietary changes and to determine progressive personal goals aimed at improving the adherence to MedDiet principles. Different tools congruent with the MI approach were used during the individual sessions to formulate dietary objectives while increasing self-determined motivation. Moreover, in accordance with the SDT [12], basic psychological needs (i.e. autonomy, competence and relatedness) were supported during the nutritional intervention via the MI approach in order to increase self-determined motivation. Briefly, the dietitian had a client-centered approach, put no pressure on participants about the dietary objectives to be chosen and no emphasis was put on body weight control. Men and women were encouraged to maintain dietary changes in an autonomous way at the end of the nutritional program and there was no additional contact with the dietitian after the end of the 12-week intervention. However, men and women were invited to participate to the follow-up period for measurements of anthropometric and metabolic parameters and completion of questionnaires. This clinical trial was registered at http://www.clinicaltrials.gov as NCT01852721.

### Measurements of dependent variables

All measurements were performed before (t = 0) and after the 12-week nutritional intervention program (t = 3 months), and then 3 and 6 months after the end of the nutritional intervention (t = 6 months and t = 9 months, respectively).

#### Dietary variables

A validated food frequency questionnaire (FFQ) [27] was administered by a registered dietitian. The FFQ is based on typical foods available in Québec and contains 91 items and 33 subquestions. Participants were questioned about the frequency of intake of different foods and drinks during the last month and could report the frequency of these intakes in terms of day, week or month. As previously described [6], the Medscore was calculated based on the FFQ and allowed to assess the level of adherence to the Mediterranean food pattern, which can vary between 0 and 44 points. Components of the Medscore are: grains (whole and refined); fruits (whole and juices); vegetables (whole and juices); legumes, nuts and seeds; olive oil (including olives); dairy products; fish (including seafoods); poultry; eggs; sweets and red meat/processed meat. Briefly, a high consumption of food groups promoted by the Mediterranean diet (bottom of the pyramid) (e.g., legumes) contributed to increase the Medscore, whereas a high consumption of food groups at the top of the Mediterranean pyramid (e.g., red meat) contributed to decrease the Medscore, as previously described [6]. Macronutrient and micronutrient intakes obtained from the FFQ were evaluated using the Nutrition Data System for Research (NDS-R, version 4.03_31) software.

#### Anthropometric and metabolic profile

Height was measured to the nearest millimeter (Seca 222 Mechanical Telescopic Stadiometer), body weight was measured to the nearest 0.1 kg (BWB-800S Digital scale, Tanita), and body mass index (BMI) was then calculated according to standardized procedures [28]. Waist circumference measure was also taken to the nearest millimeter according to standardized procedures [28] and body fat percentage was measured using the Tanita body-fat analyser, with the accuracy level being +/− 5% of the institutional standard of body composition analysis-Dual Energy X-ray Absorptiometry (DEXA) and repeatable to within +/− 1% variation when used under consistent conditions (Tanita-BC-418 body-fat analyser (Tanita Corp., Tokyo, Japan)) [29]. Blood samples were collected after a 12-hour overnight fast. Total-C, HDL-C and triglyceride concentrations in serum were measured using commercial reagents on a Modular P chemistry analyzer (with 0.8% and 1.7% of within and between assay precision, respectively) (Roche Diagnostics, Mannheim, Germany). Serum LDL-C concentrations were obtained by calculation using the Friedewald equation [30]. Plasma glucose concentrations were measured with the hexokinase enzymatic method (with 0.7% and <1.2% of within and between assay precision, respectively) and plasma insulin concentrations by electrochimiluminescence (with <2.0% and <2.8% of within and between assay precision, respectively) (Roche Diagnostics, Mannheim, Germany). Blood pressure was measured on the right arm and using an automated blood pressure monitor (BPM 300-BpTRU: Vital Signs Monitor) after 10 minutes rest in the sitting position and was computed as a mean of three readings which were highly correlated (systolic blood pressure: 0.85 ≥ r ≤0.91, *P* <0.0001; diastolic blood pressure: 0.84 ≥ r ≤0.85, *P* <0.0001).

#### Eating behaviors

Eating behaviors were assessed by the Three-Factor Eating Questionnaire (TFEQ) [31], a 51-item validated questionnaire which assesses three factors that refer to cognitions and behaviors associated with eating: dietary restraint (conscious control of food intake with concerns about shape and weight), disinhibition (overconsumption of food in response to a variety of stimuli associated with a loss of control on food intake) and hunger (food intake in response to feelings and perceptions of hunger). More specific subscales can also be derived from these three general eating behaviors [32, 33]: rigid restraint, flexible restraint, habitual susceptibility to disinhibition, emotional susceptibility to disinhibition, situational susceptibility to disinhibition, internal hunger, and external hunger.

### Statistical analyses

Dietary intakes, eating behaviors, anthropometric and metabolic variables measured at different time points are presented in tables as means ± standard deviations. When within gender changes are presented they are expressed as percentages of change from baseline values. The Student’s t-test allowed comparisons of baseline characteristics between men and women. The Chi-Square test was performed to compare the attrition rate between men and women. The PROC MIXED procedure, which allow the inclusion of participants with missing data at some time points [34] was also performed. MIXED procedures for repeated measurements were performed to determine gender, time and gender by time interactions effects on changes in dependent variables measured (delta values). Since three different dietitians were in charge of providing the intervention, the intervener effect was also tested by a MIXED procedure. Delta values were calculated as post nutritional intervention values (post-nutritional intervention minus pre-nutritional intervention values) and as follow-up values at 3-month (6 months minus pre-nutritional intervention values) and 6-month post intervention (9 months minus pre-nutritional intervention values), respectively. Using this approach, a significant time effect means that the magnitude of the change is varying with time while a non-significant time effect means that changes are maintained with time. Moreover, a significant gender by time interaction means that the trajectory of changes with time is not the same in men and women. The Lsmeans procedure, which can be defined as a linear combination (sum) of the estimated effects, e.g. means, from a linear model and based on the model used, allowed determining significant changes in outcomes over time within each gender. When significant gender by time interactions were observed, simple effects between times and gender were tested to determine precisely the location of the main interaction effect. Pairwise differences between and within gender were further tested with the Tukey-Kramer adjustment. Additional analyses were performed in order to determine if gender differences in changes in dietary intakes, eating behaviors, anthropometric and metabolic variables were still significant when accounting for the baseline value. For variables not normally distributed, a transformation was performed but these variables are presented as raw data in the tables. In order to determine sample size, we considered a difference of 35% in the change in Medscore as being clinically significant, based on results of a previous study from our group [6]. Therefore, a final sample size of 45 men and 45 women was needed to detect a gender difference of 35% in the change in Medscore with a power of 0.80 and alpha of 0.05, considering that standard deviation corresponds to 55% of the mean of the change in Medscore. The probability level for significance used for the interpretation of all statistical analyses was set at an alpha level of *P* ≤0.05. All analyses were performed using SAS statistical software (version 9.2, SAS Institute Inc., Cary, NC).

## Results

Table 1 shows characteristics of men and women at baseline in terms of their age, anthropometric variables, metabolic profile and the quality of their diet as represented by the Medscore. Men and women included in our study were of similar age, but men had higher BMI, waist circumference, total-C to HDL-C ratio and triglyceride levels than women, whereas women had higher percentage of body fat and HDL-C levels than men. As for global quality of the diet at baseline, women tended to have a higher Medscore than men. Of the 64 men and 59 premenopausal women included in our study at baseline, 89%, 78% and 69% of men and 86%, 78% and 75% of women completed the 12-week nutritional intervention program, and the 3-month and 6-month post intervention follow-up visits respectively, without significant gender differences in the attrition rate at any of the three visits. Moreover, men and women who withdrew from the study presented similar characteristics at baseline to those who completed the intervention until the end of the follow-up (not shown).

**Table 1:** **Characteristics of men and women at baseline**

	Men (n = 64)		Women (n = 59)	
	Mean	SD	Mean	SD
Medscore (arbitrary units)	22.7	4.3	24.1^¶^	3.6
Age (years)	41.0	7.9	41.8	6.7
Body mass index (kg/m^2^)	30.8	4.4	29.6*	6.0
Body fat (%)	26.7	4.5	39.2*	6.2
Waist circumference (cm)	106.1	10.2	95.8*	11.5
LDL-C (mmol/l)	3.6	0.7	3.6	0.7
HDL-C (mmol/l)	1.1	0.2	1.4*	0.3
Total-C/HDL-C ratio	5.1	1.0	4.2*	0.9
Triglycerides (mmol/l)	1.9	0.9	1.5*	0.6
Fasting glucose (mmol/l)	5.3	0.5	5.2	0.7

SD: Standard deviation.

For percentage of body fat, n = 52 men and n =48 women.

For lipid-lipoprotein variables and fasting glucose, n = 63 men and n = 58 women.

*Values significantly different between men and women (*P* ≤0.05, Student’s t*-*test procedure).

^¶^Trend for a significant difference between men and women (*P* = 0.06, Student’s t-test procedure).

### Changes in dietary intakes

Changes in dietary intakes in men and women are presented in Table 2. At the end of the intervention (t =3 months) as well as 3-month and 6-month post intervention (t = 6 months and 9 months, respectively), changes from baseline in energy density and in percentage of energy intake from lipids and carbohydrates were larger in men than in women. For these variables, changes were maintained during follow-up as no significant time effect was observed. Changes observed in dietary fiber intake and in the percentage of energy intake from saturated fatty acids were also larger in men than in women. However, for those variables, the magnitude of change decreased with time (time effects; *P* = 0.008 and *P* = 0.0002, respectively). Moreover, although significant changes in percentage of energy intake from polyunsaturated and trans fatty acids were observed in both men and women in response to the nutritional intervention, no gender differences were observed for these variables and they progressively returned towards baseline values during follow-up (time effects; *P* = 0.004 and *P* = 0.05 for percentage of energy intake from polyunsaturated and trans fatty acids, respectively). No gender by time interaction was observed for nutritional intakes, meaning that trajectories of changes during the follow-up were not significantly different between men and women.

**Table 2:** **Changes in dietary intakes in men and women**

	Men			Women			Gender effect	Time effect	Gender x time interaction	Gender effect adjusted for baseline value
Variables	Mean	SD	% Change vs. Baseline	Mean	SD	% Change vs. Baseline				
							***P***	***P***	***P***	***P***
Energy intake (kcal)							0.38	0.99	0.84	0.02
Baseline	3065	896	-	2457	571	-				
T = 3 months	2768	713	−9.7*	2335	527	−5.0				
T = 6 months	2757	752	−10.0*	2284	497	−7.0^¶^				
T = 9 months	2759	636	−10.0*	2329	470	−5.2				
Energy density (kcal/g)^£^							0.02	0.43	0.11	0.71
Baseline	1.32	0.24	-	1.21	0.17	-				
T = 3 months	1.21	0.23	−8.3*	1.19	0.17	−1.7				
T = 6 months	1.25	0.21	−5.3*	1.16	0.17	−4.1^¶^				
T = 9 months	1.26	0.21	−4.5*	1.20	0.17	−0.8				
% Carbohydrates							0.03	0.81	0.74	0.44
Baseline	42.2	5.3	-	44.5	5.8	-				
T = 3 months	44.1	5.8	4.5*	44.8	5.5	0.7				
T = 6 months	43.9	6.3	4.0*	44.9	5.4	0.9				
T = 9 months	44.4	6.0	5.2*	44.9	5.6	0.9				
Total dietary fibers (g)							0.003	0.008	0.90	0.0005
Baseline	27.2	9.4	-	25.6	6.3	-				
T = 3 months	32.8	10.8	20.6*	28.1	7.5	9.8*				
T = 6 months	30.1	10.7	10.7*	25.1	6.0	−2.0				
T = 9 months	30.6	11.9	12.5*	25.2	6.2	−1.6				
% Proteins							0.99	0.04	0.67	0.40
Baseline	18.3	2.5	-	17.8	2.8	-				
T = 3 months	18.4	2.8	0.5	17.9	2.9	0.6				
T = 6 months	18.0	3.0	−1.6	17.6	2.3	−1.1				
T = 9 months	17.9	2.6	−2.2	17.1	2.6	−3.9^¶^				
% Lipids							0.01	0.76	0.66	0.25
Baseline	36.8	4.8	-	35.1	4.5	-				
T = 3 months	34.6	5.3	−6.0*	34.6	5.2	−1.4				
T = 6 months	34.8	5.2	−5.4*	34.3	4.8	−2.3				
T = 9 months	35.1	4.9	−4.6*	35.1	4.8	0.0				
% MUFA							0.07	0.76	0.21	0.58
Baseline	15.5	2.8	-	14.6	2.7	-				
T = 3 months	15.1	2.9	−2.6	15.1	3.4	3.4				
T = 6 months	15.2	2.9	−1.9	14.5	2.4	−0.7				
T = 9 months	14.9	2.7	−3.9*	15.1	3.3	3.4				
% PUFA							0.93	0.004	0.94	0.10
Baseline	6.2	1.5	-	5.9	1.4	-				
T = 3 months	6.8	1.6	9.7*	6.3	1.4	6.8*				
T = 6 months	6.4	1.5	3.2	5.8	1.1	−1.7				
T = 9 months	6.5	1.5	4.8	5.9	1.2	0.0				
% SFA							0.002	0.0002	0.67	0.004
Baseline	12.2	2.7	-	11.8	1.7	-				
T = 3 months	10.1	2.4	−17.2*	10.5	2.0	−11.0*				
T = 6 months	10.6	2.4	−13.1*	11.4	2.5	−3.4				
T = 9 months	11.0	2.4	−9.8*	11.5	2.1	−2.5				
% Trans							0.13	0.05	0.33	0.73
Baseline	1.4	0.4	-	1.3	0.3	-				
T = 3 months	1.1	0.4	−21.4*	1.1	0.4	−15.4*				
T = 6 months	1.2	0.5	−14.3*	1.1	0.3	−15.4				
T = 9 months	1.3	0.3	−7.1*	1.1	0.3	−15.4				
% Alcohol							0.63	0.14	0.76	0.62
Baseline	2.7	2.4	-	2.7	2.3	-				
T = 3 months	2.9	2.6	7.4	2.7	2.1	0.0				
T = 6 months	3.3	3.1	22.2*	3.1	2.4	14.8				
T = 9 months	2.6	2.4	−3.7	2.9	2.8	7.4				

For T = 0, T = 3, T = 6 and T = 9 months, n =64, 57, 50, 44 men and n =59, 51, 46, 44 women, respectively.

SD: Standard deviation.

MUFA: Monounsaturated fatty acids; PUFA: Polyunsaturated fatty acids; SFA: Saturated fatty acids; Trans: Trans fatty acids.

*P ≤0.05; Significant change within the same gender.

^¶^ *P* ≤0.10; Trend for a significant change within the same gender.

^£^Including caloric foods and drinks.

As for the Medscore (Table 3), although a significant increase was observed in men and women, no gender difference was found in changes measured at the end of the nutritional intervention and at follow-up visits. However, the Medscore progressively returned towards baseline values during the follow-up in men and women as shown by the significant time effect (*P* <0.0001). Similarly, for olive oil and olives, legumes, nuts and seeds, and fish and seafood intakes, the magnitude of change decreased with time (time effects; *P* = 0.01, *P* <0.0001 and *P* = 0.004, respectively) in both men and women. On the other hand, gender differences were observed in red and processed meat and whole fruit intakes, for which changes were larger in men than in women. The magnitude of change also decreased with time for red and processed meat intake as indicated by the significant time effect (*P* = 0.0002). No gender by time interaction was observed for the Medscore and its components, meaning that trajectories of changes during the follow-up were the same in men and women. Moreover, the change in Medscore was not influenced by the dietitian in charge of the intervention as indicated by the analysis of variance (*P* = 0.68).

**Table 3:** **Changes in Medscore and food groups in men and women**

	Men			Women			Gender effect	Time effect	Gender x time interaction	Gender effect adjusted for baseline value
Variables	Mean	SD	% Change vs. Baseline	Mean	SD	% Change vs. Baseline				
							***P***	***P***	***P***	***P***
Medscore (arbitrary units)							0.25	< 0.0001	0.42	0.70
Baseline	22.7	4.3	-	24.1	3.6	-				
T = 3 months	27.6	4.7	21.6*	27.2	4.9	12.9*				
T = 6 months	25.4	4.8	11.9*	25.8	4.2	7.1*				
T = 9 months	24.6	4.6	8.4*	24.9	5.1	3.3				
Olives (portions/d)							0.77	0.01	0.15	0.96
Baseline	1.1	1.4	-	1.0	0.9	-				
T = 3 months	1.5	1.1	36.4^¶^	1.6	1.7	60.0*				
T = 6 months	1.4	1.6	27.3	1.0	0.8	0.0				
T = 9 months	1.1	0.8	0.0	1.2	1.4	20.0				
Whole fruits (portions/d)							0.04	0.18	0.22	0.68
Baseline	1.7	1.4	-	2.5	1.5	-				
T = 3 months	2.6	2.0	52.9*	2.6	1.4	4.0				
T = 6 months	2.1	1.4	23.5*	2.6	1.4	4.0				
T = 9 months	2.1	1.6	23.5*	2.6	1.3	4.0				
Whole vegetables (portions/d)							0.13	0.08	0.76	0.62
Baseline	3.8	1.9	-	4.3	1.4	-				
T = 3 months	4.3	2.1	13.2*	4.3	1.7	0.0				
T = 6 months	4.1	1.9	7.9	4.0	1.4	−7.0				
T = 9 months	3.7	1.6	−2.6	4.0	2.0	−7.0				
Legumes, nuts and seeds (portions/d)^†^							0.16	< 0.0001	0.57	0.002
Baseline	1.3	1.3	-	0.8	0.6	-				
T = 3 months	1.9	1.2	46.2*	1.2	0.6	50.0*				
T = 6 months	1.6	1.0	23.1	0.9	0.5	12.5				
T = 9 months	1.5	1.1	15.4	0.9	0.5	12.5				
Whole grain products (portions/d)							0.59	0.86	0.36	0.005
Baseline	3.2	1.7	-	2.3	1.6	-				
T = 3 months	3.7	1.4	15.6*	2.9	1.3	26.1*				
T = 6 months	3.8	2.3	18.8*	2.8	1.4	21.7^¶^				
T = 9 months	3.9	2.1	21.9*	2.7	1.3	17.4				
Refined grain products (portions/d)							0.54	0.48	0.07	0.51
Baseline	3.3	1.8	-	2.7	1.6	-				
T = 3 months	1.9	1.1	−42.4*	1.9	1.3	−29.6*				
T = 6 months	2.5	1.9	−24.2*	1.8	1.0	−33.3*				
T = 9 months	2.2	1.5	−33.3*	2.1	1.2	−22.2*				
Milk and dairy products (portions/d)							0.62	0.54	0.74	0.60
Baseline	3.1	2.1	-	2.6	1.1	-				
T = 3 months	2.8	1.8	−9.7	2.4	1.0	−7.7				
T = 6 months	2.7	1.7	−12.9	2.6	1.4	0.0				
T = 9 months	2.7	1.1	−12.9	2.6	1.2	0.0				
Poultry (portions/w)							0.94	0.24	0.83	0.12
Baseline	6.4	4.7	-	4.8	2.9	-				
T = 3 months	5.3	3.8	−17.2	4.0	2.5	−16.7				
T = 6 months	4.5	3.6	−29.7*	3.9	2.0	−18.8^¶^				
T = 9 months	5.0	3.3	−21.9	4.0	2.7	−16.7				
Fish and seafood (portions/w)							0.44	0.004	0.95	0.16
Baseline	4.5	3.5	-	3.8	2.8	-				
T = 3 months	7.5	5.6	66.7*	6.2	4.0	63.2*				
T = 6 months	6.8	5.1	51.1*	5.5	3.3	44.7*				
T = 9 months	6.2	3.7	37.8*	4.8	3.2	26.3				
Red meat/processed meat (portions/w)							0.03	0.0002	0.14	0.83
Baseline	11.6	6.6	-	8.0	4.7	-				
T = 3 months	5.6	3.8	−51.7*	4.8	3.1	−40.0*				
T = 6 months	7.8	6.0	−32.8*	5.7	3.5	−28.8*				
T = 9 months	7.1	5.3	−38.8*	5.8	3.5	−27.5*				
Eggs (portions/w)							0.21	0.35	0.51	0.01
Baseline	3.2	2.7	-	2.3	1.7	-				
T = 3 months	3.3	2.6	3.1	2.1	1.7	−8.7				
T = 6 months	3.8	3.5	18.8^¶^	2.2	1.7	−4.3				
T = 9 months	3.7	3.0	15.6	2.1	1.9	−8.7				
Sweets (portions/w)^†^							0.95	0.47	0.25	0.23
Baseline	13.8	33.4	-	9.2	9.1	-				
T = 3 months	7.3	12.3	−47.1*	6.4	6.0	−30.4				
T = 6 months	9.3	14.3	−32.6	6.5	5.5	−29.3				
T = 9 months	7.6	5.9	−44.9	8.6	9.3	−6.5				

For T = 0, T = 3, T = 6 and T = 9 months, n =64, 57, 50, 44 men and n =59, 51, 46, 44 women, respectively.

SD: Standard deviation; d: day; w: week.

*P ≤0.05; Significant change within the same gender.

^¶^ *P* ≤0.10; Trend for a significant change within the same gender.

^†^Analysis was performed on transformed values.

When dietary changes were adjusted for the baseline value of the response variable (Tables 2 and 3), gender differences remained significant for dietary fiber intake and percentage of energy intake from saturated fatty acids only. All other gender differences initially observed for dietary changes became non-significant after this adjustment. Moreover, significant gender differences were observed for legumes, nuts and seeds, whole grain products and egg intakes once adjusted for the baseline value, with greater increases observed for these variables in men than in women.

### Changes in anthropometric and metabolic variables

As shown in Table 4, whereas men had significantly lower waist circumference at the end of the intervention as well as 3-month and 6-month post intervention than at baseline, women’s waist circumference decreased in response to the intervention but returned towards baseline values during follow-up (gender by time interaction, *P* = 0.05). No significant gender differences or time effects were reported for body weight and percentage of body fat. As for metabolic variables, changes observed in total-C to HDL-C ratio, triglyceride levels and triglycerides to HDL-C ratio were significantly more pronounced in men than in women at the end of the nutritional intervention as well as at the end of follow-up. Moreover, whereas changes in total-C to HDL-C ratio, triglyceride levels and triglycerides to HDL-C ratio were maintained during follow-up, the magnitude of change observed in HDL-C levels and diastolic blood pressure tended to vary with time (time effects; *P* = 0.06 and *P* = 0.07, respectively).

**Table 4:** **Changes in anthropometric and metabolic variables in men and women**

	Men			Women			Gender effect	Time effect	Gender x time interaction
Variables	Mean	SD	% Change vs. Baseline	Mean	SD	% Change vs. Baseline			
							***P***	***P***	***P***
Body weight (kg)							0.13	0.71	0.67
Baseline	96.6	15.5	-	77.9	16.2	-			
T = 3 months	95.2	14.4	−1.4*	77.2	17.1	−0.9^¶^			
T = 6 months	94.5	12.4	−2.2*	78.1	17.5	0.3			
T = 9 months	94.2	13.3	−2.5*	77.8	17.5	−0.1			
Body fat (%)							0.12	0.86	0.82
Baseline	26.7	4.5	-	39.2	6.2	-			
T = 3 months	25.6	4.6	−4.1*	39.2	6.6	0.0			
T = 6 months	26.2	5.3	−1.9*	38.9	6.5	−0.8			
T = 9 months	25.6	5.0	−4.1*	38.4	6.4	−2.0			
Waist circumference (cm)							0.10	0.39	0.05
Baseline	106.1	10.2	-	95.8	11.5	-			
T = 3 months	104.2	9.5	−1.8*	94.5	12.6	−1.4*			
T = 6 months	103.4	8.9	−2.5*	95.0	12.6	−0.8			
T = 9 months	103.1	9.9	−2.8*	95.9	12.5	0.1			
HDL-C (mmol/l)							0.12	0.06	0.50
Baseline	1.15	0.22	-	1.44	0.30	-			
T = 3 months	1.19	0.25	3.5*	1.44	0.28	0.0			
T = 6 months	1.20	0.25	4.3*	1.41	0.28	−2.1			
T = 9 months	1.22	0.24	6.1*	1.46	0.31	1.4*			
LDL-C (mmol/l)							0.22	0.86	0.15
Baseline	3.64	0.69	-	3.65	0.69	-			
T = 3 months	3.70	0.64	1.6	3.60	0.62	−1.4			
T = 6 months	3.61	0.65	−0.8^¶^	3.71	0.76	1.6			
T = 9 months	3.58	0.71	−1.6	3.73	0.66	2.2			
Total-C/HDL-C ratio							0.0007	0.18	0.25
Baseline	5.08	1.01	-	4.16	0.86	-			
T = 3 months	4.87	0.99	−4.1*	4.06	0.84	−2.4			
T = 6 months	4.80	0.97	−5.5*	4.22	0.79	1.4			
T = 9 months	4.60	0.85	−9.4*	4.13	0.78	−0.7			
Triglycerides (mmol/l)							0.03	0.09	0.97
Baseline	1.89	0.85	-	1.50	0.58	-			
T = 3 months	1.60	0.66	−15.3*	1.36	0.45	−9.3			
T = 6 months	1.64	0.70	−13.2*	1.46	0.51	−2.7			
T = 9 months	1.61	0.98	−14.8*	1.42	0.52	−5.3			
Triglycerides/HDL-C ratio							0.02	0.14	0.98
Baseline	1.77	0.99	-	1.10	0.49	-			
T = 3 months	1.44	0.76	−18.6*	0.99	0.42	−10.0			
T = 6 months	1.49	0.86	−15.8*	1.07	0.44	−2.7			
T = 9 months	1.47	1.39	−16.9*	1.02	0.42	−7.3			
Systolic blood pressure (mm Hg)							0.76	0.79	0.27
Baseline	119.78	14.60	-	109.22	11.24	-			
T = 3 months	119.28	10.63	−0.4	109.51	11.72	0.3			
T = 6 months	118.53	11.50	−1.0	111.17	11.61	1.8			
T = 9 months	120.36	10.02	0.5^¶^	109.26	8.96	0.04			
Diastolic blood pressure (mm Hg)							0.52	0.07	0.90
Baseline	75.59	9.47	-	70.76	7.92	-			
T = 3 months	72.40	8.48	−4.2*	68.94	8.17	−2.6			
T = 6 months	73.97	8.97	−2.1	70.19	9.15	−0.8			
T = 9 months	72.32	7.95	−4.3*	69.04	6.96	−2.4			
Fasting glucose (mmol/l)							0.97	0.39	0.84
Baseline	5.27	0.55	-	5.16	0.69	-			
T = 3 months	5.28	0.41	0.2	5.21	0.68	1.0			
T = 6 months	5.30	0.43	0.6	5.29	1.19	2.5			
T = 9 months	5.38	0.52	2.1^¶^	5.17	0.59	0.2			
Fasting insulin (pmol/l)							0.99	0.41	0.05
Baseline	100.48	44.99	-	88.53	45.36	-			
T = 3 months	98.95	44.34	−1.5	88.22	40.27	−0.4			
T = 6 months	98.70	50.04	−1.8	83.11	36.47	−6.1			
T = 9 months	85.77	34.17	−14.6	84.25	38.16	−4.8			

For T = 0, T = 3, T = 6 and T = 9 months, n =64, 57, 50, 44 men and n =59, 51, 46, 44 women, respectively.

For percentage of body fat, T = 0, 3, 6 and 9 months: n =52, 47, 50, 45 men and n =48, 41, 44, 43 women, respectively.

For lipid-lipoprotein variables, T = 0, 3, 6 and 9 months: n =63, 56, 48, 41 (n =40 LDL-C) men and n =58, 51, 44, 44 women.

For fasting glucose and insulin, T = 0, 3, 6 and 9 months: n =64, 57, 50, 44 men and n =58, 51, 44, 44 women.

SD: Standard deviation.

*P ≤0.05; Significant change within the same gender.

^¶^ *P* ≤0.10; Trend for a significant change within the same gender.

### Changes in eating behaviors

Table 5 shows changes in eating behaviors in men and women in response to the 12-week nutritional intervention and at 3-month and 6-month post intervention. Overall, men showed a significant greater decrease in habitual susceptibility to disinhibition than women and a trend for a more pronounced increase in dietary restraint in men than in women was observed. Moreover, changes reported in eating behaviors in men and women were all maintained through the end of the follow-up (no significant time effect).

**Table 5:** **Changes in eating behaviors in men and women**

	Men			Women			Gender effect	Time effect	Gender x time interaction
Variables	Mean	SD	% Change vs. Baseline	Mean	SD	% Change vs. Baseline			
							***P***	***P***	***P***
Dietary restraint							0.07	0.88	0.83
Baseline	5.9	3.2	-	6.9	4.1	-			
T = 3 months	7.4	3.9	25.4*	7.6	3.9	10.1^¶^			
T = 6 months	8.0	4.7	35.6*	7.3	3.3	5.8			
T = 9 months	8.4	5.2	42.4*	7.4	3.5	7.2^¶^			
Flexible restraint							0.10	0.41	0.55
Baseline	1.9	1.4	-	2.4	1.6	-			
T = 3 months	2.4	1.5	26.3*	2.8	1.6	16.7			
T = 6 months	2.7	1.7	42.1*	2.8	1.4	16.7^¶^			
T = 9 months	3.0	1.9	57.9*	2.8	1.5	16.7			
Rigid restraint							0.43	0.88	0.19
Baseline	1.7	1.3	-	1.8	1.7	-			
T = 3 months	1.8	1.5	5.9	1.9	1.6	5.6			
T = 6 months	2.2	1.9	29.4*	1.8	1.4	0.0			
T = 9 months	2.2	1.8	29.4*	1.9	1.5	5.6			
Disinhibition							0.11	0.58	0.42
Baseline	6.0	2.8	-	6.2	2.8	-			
T = 3 months	4.9	2.3	−18.3*	5.3	3.0	−14.5^¶^			
T = 6 months	4.9	2.2	−18.3*	5.3	2.6	−14.5			
T = 9 months	4.8	2.2	−20.0*	5.4	3.0	−12.9			
Habitual susceptibility to disinhibition							0.03	0.75	0.39
Baseline	0.7	1.0	-	0.7	1.0	-			
T = 3 months	0.4	0.8	−42.9*	0.6	1.0	−14.3			
T = 6 months	0.4	0.6	−42.9*	0.4	0.8	−42.9			
T = 9 months	0.2	0.4	−71.4*	0.4	0.8	−42.9			
Situational susceptibility to disinhibition							0.09	0.77	0.80
Baseline	3.0	1.4	-	2.5	1.5	-			
T = 3 months	2.3	1.3	−23.3*	2.0	1.5	−20.0^¶^			
T = 6 months	2.3	1.5	−23.3*	2.1	1.4	−16.0			
T = 9 months	2.2	1.5	−26.7*	2.1	1.6	−16.0			
Emotional susceptibility to disinhibition							0.23	0.22	0.75
Baseline	0.7	1.0	-	1.6	1.2	-			
T = 3 months	0.7	1.0	0.0	1.2	1.2	−25.0*			
T = 6 months	0.7	0.9	0.0	1.2	1.2	−25.0			
T = 9 months	0.7	1.0	0.0	1.2	1.2	−25.0			
Hunger							0.32	0.81	0.64
Baseline	4.8	3.0	-	4.2	2.8	-			
T = 3 months	3.3	2.4	−31.3*	3.0	2.7	−28.6*			
T = 6 months	3.5	2.7	−27.1*	3.0	2.5	−28.6*			
T = 9 months	3.4	3.0	−29.2*	3.2	2.8	−23.8			
Internal hunger							0.53	0.68	0.35
Baseline	1.9	1.8	-	1.6	1.6	-			
T = 3 months	1.2	1.4	−36.8*	0.9	1.4	−43.8*			
T = 6 months	1.3	1.5	−31.6*	1.0	1.6	−37.5^¶^			
T = 9 months	1.2	1.7	−36.8*	1.1	1.6	−31.3			
External hunger							0.57	0.66	0.12
Baseline	1.9	1.5	-	1.8	1.4	-			
T = 3 months	1.1	1.3	−42.1*	1.4	1.3	−22.2			
T = 6 months	1.4	1.4	−26.3^¶^	1.1	1.1	−38.9*			
T = 9 months	1.4	1.4	−26.3*	1.4	1.2	−22.2			

For T = 0, T = 3, T = 6 and T = 9 months, n =63, 57, 49, 44 men and n =59, 50, 45, 44 women, respectively.

In men, T = 0: flexible restraint and internal hunger, n =60 and external hunger, n =62; T = 3 months: dietary restraint, flexible restraint and rigid restraint, disinhibition and habitual susceptibility to disinhibition, internal and external hunger, n =56; T = 6 months: flexible restraint and external hunger, n =48; T = 9 months: habitual susceptibility to disinhibition and external hunger, n =43.

In women, T = 0: flexible restraint, habitual susceptibility to disinhibition, internal and external hunger, n =58 and rigid restraint, n =57; T = 3 months: flexible restraint and internal and external hunger, n =49 and rigid restraint, n =48; T = 6 months: flexible restraint and habitual susceptibility to disinhibition, n =43 and rigid restraint and external hunger, n =44; T = 9 months: rigid restraint, n =43.

SD: Standard deviation.

* *P* ≤0.05; Significant change within the same gender.

^¶^ *P* ≤0.10; Trend for a significant change within the same gender.

## Discussion

Our study aimed to document gender differences in long-term effects of a 12-week nutritional intervention program promoting the adoption of the MedDiet and based on the SDT on dietary intakes, eating behaviors, anthropometric and metabolic variables, in men and women presenting risk factors for CVD. Overall, our results showed that most of the gender differences found in dietary intakes and metabolic profile could be explained by gender differences in changes occurring during the 12-week intervention since similar levels of maintenance of changes were generally observed in men and women during the post-intervention period.

No gender difference was observed in the change in adherence to the MedDiet measured at the end of the follow-up period. However, gender differences were observed for specific components of the MedDiet, for which more pronounced changes were found in men than in women. Those changes were also concordant with gender differences observed in changes in some nutrient intakes. For example, fruits are recognized for their high fiber content and accordingly, greater increases in fruit as well as in dietary fiber intakes were observed in men than in women. Similarly, greater decreases observed in red and processed meat consumption in men than in women are concordant with greater decreases in saturated fatty acids intake also observed in men. The fact that these dietary changes were more pronounced in men at the end of follow-up appears to be explained by the greater amplitude of change observed in dietary intakes in men than women during the 12-week nutritional intervention. In this regard, women had baseline dietary habits that were more concordant with principles of the MedDiet, which might explain why they did not improve as much as men, due to a ceiling effect [35]. This hypothesis is well supported by the fact that many gender differences in dietary changes were no longer significant once adjusted for the baseline value of the response variable. However, some differences between men and women remained significant despite this adjustment for the baseline value, suggesting that other factors than baseline diet’s characteristics might also explain why dietary changes in response to the 12-week nutritional intervention were not as important in women as in men. Accordingly, a previous study has shown that more men than women consider that not knowing how to take preventive action for CVD was a barrier to cardiovascular health [36]. In line with these previous observations, it is possible that our intervention was more efficient to overcome barriers more typically identified by men. In fact, our intervention aimed to support autonomy and competence, especially by improving nutrition knowledge and skills according to individuals’ needs in order to take action and apply relevant strategies related to dietary changes. In addition, although the literature provides a limited understanding of the contexts of help seeking in health prevention in men, it seems that self-reliance and autonomy remain important for them [37, 38]. Therefore, it can be speculated that men might feel more comfortable to choose their own dietary objectives and strategies than women and that the nature of support offered in our program was more in congruence with men’s needs for the support of autonomy and competence in the context of food regulation, as suggested by their greater improvements in overall dietary intakes. In sum, individual characteristics and intervention components may have contributed to gender differences observed in the success of men and women in long-term dietary changes and would warrant to be further considered in the future.

Our results suggest that men and women showed similar limits in maintaining dietary changes in the longer term. The fact that the study was conducted in a non-Mediterranean country could explain some of the results obtained, as supported by Hoffman and Gerber [39]. In fact, when examining the trajectory of changes of the components of the MedDiet, it can be noticed that intakes of food group that returned towards baseline values are generally not considered as the most familiar foods for Quebecers (e.g., legumes and fish intakes) [40, 41] or represent a staple in the diet (e.g., red and processed meat intake) [42]. A greater emphasis in terms of nutritional education (e.g., alternatives choices for more traditional foods, nutritional value of unfamiliar foods) and culinary skills specifically related to those food groups during the intervention program might have contributed to a better maintenance of changes in the long-term. Moreover, a greater emphasis by the dietitian towards the development of a social network among participants during group sessions could have also been beneficial for men and women as indicated in the literature [43, 44]. In our study, no additional contact was provided after the end of the 12-week nutritional education program as we wanted to assess ability of men and women to maintain adherence to the MedDiet principles in an autonomous way. However, according to the literature, it is possible that success in long-term diet adherence could have been improved by offering a program of a lesser intensity but provided on a longer period of time [45].

In addition to gender differences observed in dietary intakes, men and women were different with regard to eating behaviors. In fact, men showed a greater decrease in habitual susceptibility to disinhibition than women. Although the focus of the nutritional intervention program was not on changing eating behaviors, results suggest that more pronounced dietary changes observed in men in response to the intervention can have led to changes in the regulation of their eating behaviors. Accordingly, we found that an increase in the Medscore was associated with lower levels of disinhibition in men (data not shown). Previous studies indicate that different components of the MedDiet seem to modulate the brain serotonin pathway [46, 47], and findings support the association between dysregulation of the serotonergic systems and eating disorders [48]. Moreover, a recent study reported that individuals with higher level of adherence to the MedDiet had lower score for binge eating disorder [49]. In line with our results, it can thus be hypothesized that adherence to the MedDiet pattern could have contributed to improvements in eating behaviors in men, in part through neurobiological mechanisms. In addition, as supported by the literature, the high satiating properties of the MedDiet [50, 51] may also have contributed to the beneficial impact of the MedDiet on men’s eating behaviors regulation.

As for anthropometric profile, differences in trajectories of changes with time were observed between men and women for waist circumference. In agreement with a previous study [10], improvements in the quality of dietary intakes could have been beneficial for anthropometric changes in men through a negative energy imbalance. In this regard, the fact that dietary intakes were self-reported and thus subjected to different form of bias including underreporting of energy intake which is more common in women than in men [52] might explain why we did not detect significant gender differences in the reduction of energy intake in response to the intervention. Furthermore, it is possible that some sex differences could also have contributed to differences observed in changes in waist circumference between men and women. Studies have shown that men are more likely than women to experience a preferential mobilization of abdominal fat in response to lifestyles changes [53, 54]. In addition, changes in eating behaviors observed in men can also have contributed to improve their anthropometric profile. Indeed, disinhibition has been reported as a strong predictor of body weight gain [55], and in agreement our results indicate that men with greater decreases in disinhibition levels tended to have more important decreases in waist circumference and body weight at follow-up (data not shown).

In accordance with the more important changes in dietary variables and waist circumference observed in men than in women in response to the intervention, more pronounced decreases in risk factors for CVD were noted in men than in women and were maintained during follow-up. These results are supported by a review paper [56] reporting that a decrease in abdominal obesity is associated with improvements in risk factors for CVD. Globally, sustained metabolic changes observed in response to the intervention in men suggest that focusing on the quality of the diet can favour a decrease in daily energy intake and a decrease in waist circumference, and can thus be beneficial for optimal management of CVD risk factors, more particularly in men.

This study has important clinical implications that need to be mentioned. The fact that changes in dietary intakes as well as in the anthropometric and metabolic profile observed during the 12-week nutritional intervention are determinant of overall long-term changes underlines the importance for health professionals to target efficient and adapted nutritional approaches. Moreover, a better response to our nutritional intervention program was observed in men than in women, which indicates the need to clearly identify facilitating factors and barriers in approaches favouring men and women’s active implication in the process of adoption of healthy dietary changes. In this regard, consideration of qualitative data from focus group with men and women separately would be a promising avenue. As a perspective, it would be interesting to assess the impact of this nutritional intervention program in populations of men and women presenting less healthy dietary intakes before the nutritional intervention. Although the focus of the present study was to assess gender differences in response to an intervention promoting the Mediterranean diet, future studies could also add a control group to further document dietary and metabolic effects of similar nutritional interventions. Finally, considering the actual context of health prevention, it would also be relevant to assess outcomes from a similar nutritional education program but of a lower intensity (i.e. less time-consuming for individuals and requiring less financial resources) and using technologies such as the Web as part of the counselling sessions.

Overall, these results indicate that the response to the 12-week nutritional intervention program based on the Self-Determination Theory leads to more pronounced beneficial changes on long-term dietary intakes in men than in women, contributing to greater improvements in metabolic profile in men.

## Abbreviations

MedDiet: Mediterranean diet
CVD: Cardiovascular diseases
SDT: Self-Determination Theory
C: Cholesterol
FSH: Follicle-stimulating hormone
Medscore: Mediterranean score
MI: Motivational interviewing
FFQ: Food frequency questionnaire
BMI: Body mass index
TFEQ: Three-Factor Eating Questionnaire.
